# Spectrophotometric Fluoride Determination Using St. John’s Wort Extract as a Green Chromogenic Complexant for Al(III)

**DOI:** 10.1021/acsomega.2c06048

**Published:** 2022-11-29

**Authors:** Batuhan Yardımcı, Ayşem Üzer, Reşat Apak

**Affiliations:** † Science and Technology Application and Research Center (ARTMER), Zonguldak Bülent Ecevit University, Kozlu, 67600 Zonguldak, Turkey; ‡ Department of Chemistry, Faculty of Engineering, Istanbul University-Cerrahpaşa, Avcilar, 34320 Istanbul, Turkey; § Turkish Academy of Sciences (TUBA), Bayraktar Neighborhood, Vedat Dalokay St. No. 112, Çankaya, 06690 Ankara, Turkey

## Abstract

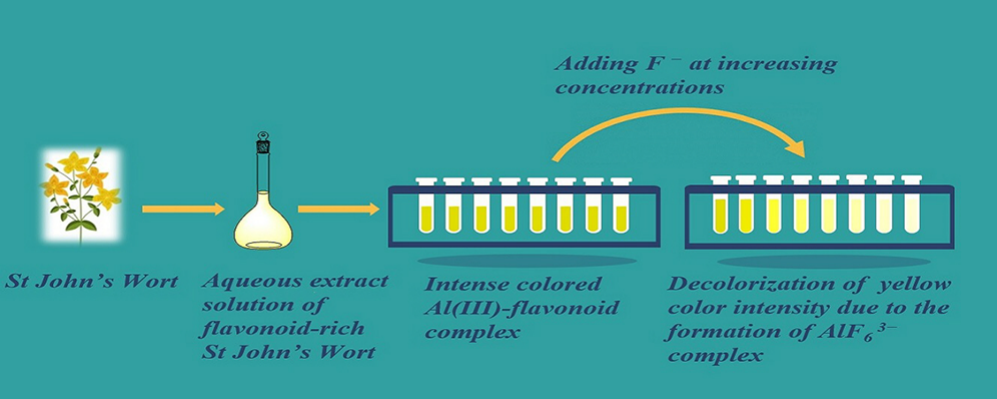

In this study, we applied an innovative approach of green analytical chemistry to develop a novel and eco-friendly chromogenic agent for fluoride determination by making use of the nontoxic Al(III)-flavonoid complex in a natural extract from St. John’s wort plant. The initial intensely yellow-colored Al(III)-flavonoid complex formed in the plant extract was converted to a colorless AlF_6_^3–^ complex with increasing amounts of fluoride, and color bleaching of the Al-flavonoid chromophore (measured as absorbance decrement) was proportional to fluoride concentration. The developed method gave a linear response within the F^–^ concentration range of 0.11–1.32 mM with the LOD and LOQ values of 0.026 mM (0.5 mg L^–1^) and 0.079 mM (1.5 mg L^–1^), respectively. The LOD value for fluoride was below the WHO-permissible limit (1.5 mg L^–1^) and the US-EPA-enforceable limit (4 mg L^–1^) in water. The possible interference effects of common anions (Cl^–^, Br^–^, I^–^, NO_3_^–^, HCO_3_^–^, SO_4_^2–^, and PO_4_^3–^) and cations (K^+^, NH_4_^+^, Ag^+^, Ca^2+^, Mg^2+^, Mn^2+^, Fe^2+^, and Fe^3+^) were investigated; the observed interferences from Fe^2+^, Fe^3+^, and PO_4_^3–^ were easily eliminated by masking iron with the necessary amount of Na_2_EDTA without affecting the blank absorbance of the Al(III)-flavonoid complex, precipitating phosphate with Ag(I) salt, and partly neutralizing alkaline water samples to pH 4 with acetic acid. The developed method was applied to real water samples and also validated against a reference spectroscopic method at the 95% confidence level.

## 1 Introduction

Healthy and safe water does not contain disease-causing microorganisms and toxic substances but instead contains macro-, micro-, and trace minerals, which are necessary for the human body in a balanced way. Water and health are directly related to each other. One of the important parameters of water quality is the amount of fluoride. Fluorine is the 13th most abundant element in the world and cannot be freely found in the environment unless it combines with other substances to form fluoride. Fluorides can be classified such as ionizable/non-ionizable and organic/inorganic. Organic fluorides do not dissolve as rapidly in water as inorganic fluoride ions not having a chemical reaction with the solvent.^1^ Fluoride, as a trace ion in water, is necessary for the growth and development of some organs, especially teeth and bones, when taken into the body at appropriate concentrations (in the range of 0.7–1.2 mg L^–1^).^2^ Industrial sectors such as the aluminum industry, oil refineries, steel production, coal processing plants, glass processing, ceramic factories, brickworks and phosphate fertilizer production, and pesticides in agricultural activities are responsible for the excess amount of human-related fluoride contamination.^3^ Consumption of fluoride in high concentrations causes dental and skeletal fluorosis,^4^ parathyroid gland damage,^5^ neurological disorders,^6^ and cardiovascular problems.^7^ Determination of fluoride concentration has been one of the important issues researched by analytical chemists because of the concentration range limitation in drinking water in terms of human health.^8^

The traditional analytical methods for the determination of fluoride ions in water are ion chromatography (IC),^9^ gas chromatography (GC),^10^ ICP-MS,^11^ AAS (i.e., by depression of Mg absorption),^12^ and ion-selective electrode-based potentiometry (ISE).^13,14^ Among these methods of F^–^ determination, ISE and spectrophotometry stand out to give satisfactory results.^15^ The disadvantages of the ISE method were reported as limited selectivity, poor precision, long equilibration time, electrode drifting, and low solubility of the lanthanum fluoride membrane crystal^16−19^ when determining fluoride at low concentrations. In addition, the ion activity, presence of interfering ions and colloidal particles, and color and temperature of solution medium may cause problems on the ISE method.^20^ Chromatographic methods for the determination of fluoride are expensive and time-consuming and require skilled specialists.^21^ Spectrophotometry is a versatile and good alternative technique for determining the concentration of inorganic ions in water samples, having many advantages such as low cost, ease of applicability, fast analysis, reliability, high sensitivity at low concentrations, and wide analytical working range.^22^ In spectrophotometric methods, dyestuff-metal complexes,^23−26^ nanoparticles,^27,28^ or synthesized organic molecules^29,30^ have been studied as the three kinds of analytical probes. However, it is known that lots of dyestuffs are both toxic and carcinogenic.^31^ Most dyes, especially synthetic ones, are nondegradable due to their stability to light and oxidants.^32^ On the other hand, considering the nanoparticle-based syntheses, some of the reducing agents used to synthesize nanoparticles may be toxic, high-priced, of low reducing capability, and may bear a contamination risk (as they can bring other impurities to the system).^33^ The synthesis of organic molecules as a fluoride receptor requires both a long time and organic solvents, limiting the use of these analysis systems in aqueous solutions.^34^ Organic solvents cause formation of hazardous wastes and also constitute a major source of volatile organic compound (VOC) emission, threatening human health.^35,36^ The US-EPA declared that to overcome the problem of disposal of hazardous wastes used in academic studies, attention should be given to the reduction of hazardous wastes.^37^ The usage of naturally sourced reagents like plant compounds instead of synthetic toxic chemicals is one of the ways to apply sustainable development principles in analytical laboratories.^38^ Lately, it has become popular to approach green analytical chemistry by enhancing currently applied methods and/or developing new methods using eco-friendly materials.^39^ Flavonoids are plant-derived polyphenolic compounds having many favorable biochemical properties.^40^ St. John’s wort (*Hypericum perforatum* L.) is one of the flavonoid-rich plants^41^ and is conventionally consumed as a herbal tea and nutritional supplement due to its remarkable bioactive properties.^42^ Flavonoids have the ability to form colored complexes with metal ions due to their carbonyl and hydroxyl groups arranged in a special (usually chelating) geometry. Metal ion coordination by flavonoids causes significant differences in certain properties, e.g., color, fluorescence, oxidation state, catalytic ability, stability, and toxicity, explaining the wide use of flavonoids in analytical chemistry, photochemistry, medicinal chemistry, and textile dyeing.^40,43^ In addition, a method based on complexing flavonoids with aluminum(III) is used to find the total flavonoid content of some species.^44^ Pękal and Pyrzynska^45^ studied and compared two common spectrophotometric procedures, named procedure 1 and procedure 2, to determine the “total flavonoid content” of food and medicinal plant samples. Method 1 involved the measurement of flavonols and flavone luteolin at 410–430 nm after addition of (only) AlCl_3_ solution, whereas method 2 investigated the same Al(III)-complexation procedure in the presence of NaNO_2_ in alkaline medium and was found specific for rutin, luteolin, and catechins but also phenolic acids at an analytical wavelength of 510 nm. Although these two procedures yielded a different order of flavonoid content for the studied plant extracts (i.e., St. John’s wort, green tea, black tea, fruit tea, chamomile, red wine, orange juice, and apple juice), St. John’s wort had the highest total flavonoid content with respect to (wrt) procedure 1 and one of the three high ones wrt procedure 2.^45^ In addition, rutin was found as the main flavonoid constituent of aqueous and different solvent extracts of St. John’s wort.^46^ It is also known that compared to the highly colored Al-flavonoid complex, Al(III) easily forms a colorless AlF_6_^3–^ complex with fluoride.^23^ With this background, we devised a readily available, nontoxic, economical, and flavonoid-rich aqueous extract (organic solvent-free) from St. John’s wort as a natural alternative chromogenic reagent for fluoride when complexed with Al(III), because the color of the Al(III)-flavonoid complex could be bleached by fluoride (due to the formation of the stable hexafluoroaluminate(III) complex) in a concentration-dependent manner, thereby enabling a selective, eco-friendly, and accurate determination of fluoride.

## 2 Methods

### 2.1 Chemicals

All reagents were of analytical reagent grade. Dried St. John’s wort was obtained from a local market. Sodium fluoride (NaF) was purchased from La Chema, Czech Republic. Aluminum nitrate nonahydrate (Al(NO_3_)_3_·9H_2_O) and ethylenediaminetetraacetic acid disodium salt dihydrate (Na_2_EDTA) were purchased from Sigma-Aldrich. Methyl salicylate (99.2% pure, 2-(HO)C_6_H_4_CO_2_CH_3_) was obtained from JQC (Huayin) Pharmaceutical Co., Ltd., and sodium acetate (CH_3_COONa), acetic acid (CH_3_COOH), ethanol, iron(III) nitrate nonahydrate (Fe(NO_3_)_3_·9H_2_O), iron(II) chloride tetrahydrate (FeCl_2_·4H_2_O), manganese(II) nitrate tetrahydrate (Mn(NO_3_)_2_·4H_2_O), ammonium chloride (NH_4_Cl), potassium nitrate (KNO_3_), calcium nitrate tetrahydrate (Ca(NO_3_)_2_·4H_2_O), magnesium nitrate hexahydrate (Mg(NO_3_)_2_·6H_2_O), sodium nitrate (NaNO_3_), sodium chloride (NaCl), sodium bromide (NaBr), sodium sulfate (Na_2_SO_4_), and boric acid (H_3_BO_3_) were purchased from Merck. Sodium phosphate monobasic (NaH_2_PO_4_) was obtained from Riedel-de Haen.

### 2.2 Apparatus

A Rayleigh VIS-723G visible spectrophotometer and its glass cuvettes (optical thickness, 5 mm) were used for all absorbance measurements. A Precisa XB 220A Analytical Balance was used to weigh all the chemicals, and a Glassco 710 DNAG hot plate with a magnetic stirrer was used to boil the ultrapure water required for the aqueous extract of St. John’s wort. A Wisetherm-fuzzy control, wısd HB-48 dry bath was used to find the optimal temperature for the recommended method.

### 2.3 Preparation of Solutions

Stock solutions of Al^3+^ and F^–^ were prepared at 1000.0 mg L^–1^ in ultrapure water and stored at +4 °C. Working solutions of different initial concentrations of F^–^ (12.5–150.0 mg L^–1^) were freshly prepared. The initial concentration of Al(III) solution at 250.0 mg L^–1^ was prepared by diluting 1000.0 mg L^–1^ stock solution for the proposed method. Acetic acid-sodium acetate buffer (pH 4.0) was prepared by mixing 0.1 M CH_3_COOH and 0.1 M CH_3_COONa solutions at an appropriate ratio.

The stock solutions of common water anions (Cl^–^, Br^–^, I^–^, NO_3_^–^, HCO_3_^–^, SO_4_^2–^, and PO_4_^3–^) and cations (K^+^, NH_4_^+^, Ag^+^, Ca^2+^, Mg^2+^, Mn^2+^, Fe^2+^, and Fe^3+^) were prepared separately at initial 75.0 g L^–1^ concentrations except HCO_3_^–^ and then mixed with F^–^ to be at several-fold (i.e., 1-, 30-, 40-, 100-, 250-, and 500-fold) of analyte in ultrapure water. Only bicarbonate (HCO_3_^–^) was prepared at an initial concentration of 50 g L^–1^ due to its limited solubility.

To apply the UV–Vis reference method, 2 × 10^–2^ M Fe^3+^, 1.0% methyl salicylate, and 625.0 mg L^–1^ F^–^ stock solutions were prepared. After 0.2020 g of Fe(NO_3_)_3_·9H_2_O was dissolved in the mixture of 1.75 mL of ultrapure water and 1.25 mL of nitric acid (1 M), the total volume was completed to 25.0 mL with ultrapure water for preparing 2 × 10^–2^ M Fe^3+^ solution. To prepare a 1.0% methyl salicylate solution, 0.5 g of methyl salicylate was weighed in a 50.0 mL volumetric flask and the total volume was completed with pure ethanol. The final concentrations of the working solutions of F^–^ to generate the calibration curve in the UV–Vis reference method were set by adding different volumes (i.e., 20.0–120.0 μL) of 625.0 mg L^–1^ stock solution to be 5.0, 10.0, 15.0, 20.0, 25.0, and 30.0 mg L^–1^.

### 2.4 Preparation of St. John’s Wort Extract Solution as a Chromogenic Agent for the Recommended Method for Fluoride Detection

**Scheme 1:**
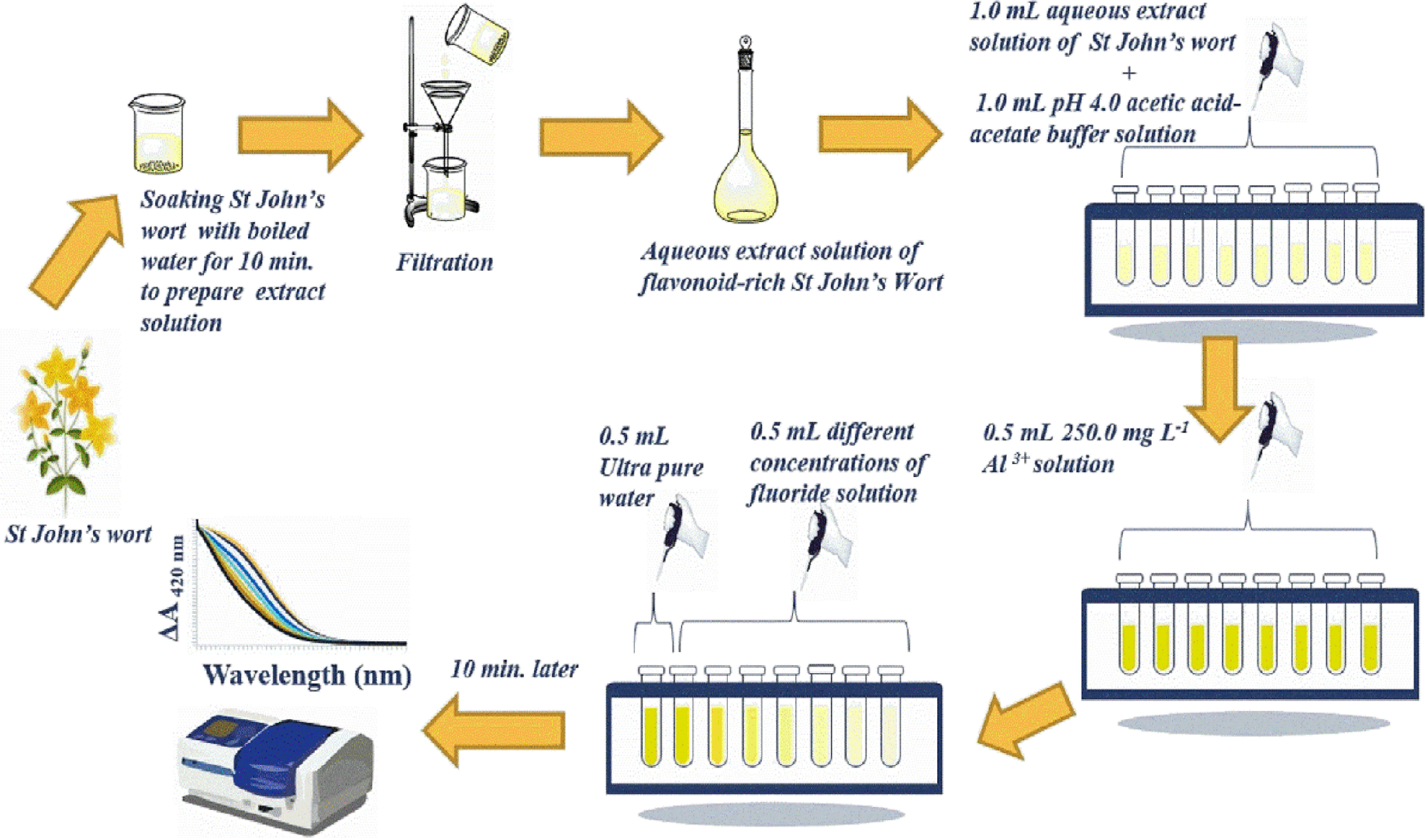
Preparation of St. John’s Wort Aqueous Extract as a Natural and Green Chromogenic Agent Followed by Al(III) Addition and the Recommended Routine Method for Fluoride Determination

A volume of 100.0 mL of freshly boiled ultrapure water was added to carefully weigh 2.0 g of dried St. John’s wort in a glass beaker. It was filtered into a 100.0 mL volumetric flask through a filter paper after waiting for 10.0 min. The final filtrate volume was completed to 100.0 mL with ultrapure water to prevent volume loss. The preparation of St. John’s wort aqueous extract as a natural and green chromogenic agent followed by Al(III) addition and the recommended method for fluoride detection are shown in Scheme 1.

A volume of 1.0 mL of St. John’s wort extract, 1.0 mL of pH 4.0 acetic acid-acetate buffer solution (0.1 M), 0.5 mL of 250.0 mg L^–1^ (initial conc.) Al^3+^ solution, and 0.5 mL of F^–^ solution at initially different concentrations (12.5–150.0 mg L^–1^) were added to the test tube in this order. For the blank solution, 0.5 mL of ultrapure water was added instead of fluoride. After waiting for 10.0 min at room temperature (RT, 25.0 °C), the absorbance values of the solutions were recorded at 420 nm wavelength against water.

Summarized procedure: for sample solutions, add 1.0 mL of St. John’s wort extract + 1.0 mL of pH 4.0 acetic acid-acetate buffer solution (0.1 M) + 0.5 mL of 250.0 mg L^–1^ (initial conc.) Al^3+^ solution + 0.5 mL of F^–^ at initially different concentrations (12.5–150.0 mg L^–1^), wait for 10.0 min at RT, and measure the absorbance at λ = 420 nm (*A*_420 nm_) against water.

For blank solution, add 1.0 mL of St. John’s wort extract + 1.0 mL of pH 4.0 acetic acid-acetate buffer solution (0.1 M) + 0.5 mL of 250.0 mg L^–1^ (initial conc.) Al^3+^ solution + 0.5 mL of ultrapure water, wait for 10.0 min at RT, and measure *A*_420 nm_ against water (*V*_total_ = 3.0 mL).

### 2.5 Investigation of Possible Interferences of Common Ions

The recovery values of fluoride were calculated by applying the recommended method in the presence of anions (Cl, Br^–^, I^–^, NO_3_^–^, HCO_3_^–^, SO_4_^2–^, and PO_4_^3–^) and cations (K^+^, NH_4_^+^, Ag^+^, Ca^2+^, Mg^2+^, Mn^2+^, Fe^2+^, and Fe^3+^). The interferences of Fe^2+^, Fe^3+^, and PO_4_^3–^ could be easily eliminated.

First, the maximum amount of Na_2_EDTA that will not dissociate the Al(III)-flavonoid complex in the recommended method was determined. If an EDTA optimization was not carried out, an excess of EDTA could decolorize the Al(III)-flavonoid complex as the target probe for fluoride attack. For this purpose, Na_2_EDTA solutions were prepared at initial concentrations of 100, 200, 300, and 400 mg L^–1^. Each Na_2_EDTA solution was added instead of fluoride in the proposed method, and ultrapure water was added instead of fluoride for the blank solution. Then, different mass ratios of Na_2_EDTA were tested along with iron ions (having different valencies) in 1:1 iron:fluoride solutions to remove the interference due to Fe^2+^ and Fe^3+^ ions by masking.

The interference effect of phosphate was eliminated using Ag^+^ ions as a precipitation agent in acidic medium. For this purpose, 0.5 mL of 1000 mg L^–1^ F^–^, 0.5 mL of 1000 mg L^–1^ PO_4_^3–^, and 0.5 mL of 1000 mg L^–1^ Ag^+^ were mixed. After the volume of the mixture was made up to 4.0 mL with ultrapure water, 0.2 mL of 0.1 mol L^–1^ HCl was added and the solution became cloudy; ultrapure water was added for dilution to a final volume of 5.0 mL, followed by keeping of the mixture in a centrifuge device at 10,000 rpm for 10 min and filtration. NaOH solution (0.1 M) was added dropwise until the pH value of the supernatant phase was 3.2 to 4.0; the supernate was diluted to 10.0 mL with ultrapure water.

### 2.6 Application of the Recommended Method to Real Water Samples

The recommended method was applied to mineral water and artificial wastewater samples. Then, the recovery (%) and RSD (%) values of fluoride were recorded. For the application of the recommended method to real water samples, the necessary preprocesses for the determination of fluoride in mineral water were carried out as follows: Initially, the pH was adjusted to 4.0 by adding 78 μL of concentrated acetic acid (17.4 M) to 20.0 mL of mineral water. Solutions were prepared in a total volume of 5.0 mL by adding certain volumes (0–1.0 mL) of fluoride at an initial concentration of 625.0 mg L^–1^ to each 5.0 mL volumetric flask containing 4.0 mL of mineral water at pH set to 4.0 (*V*_total_ = 5.0 mL). If necessary, the total volume was completed to 5 mL with ultrapure water.

The prepared artificial wastewater was diluted to have the initial concentrations of 50.0 mg L^–1^ F^–^ and 16.7 mg L^–1^ B (H_3_BO_3_ was used as a boron source). Later, solutions were prepared with a total volume of 4.0 mL by adding certain volumes (0.1–0.5 mL) of fluoride at an initial concentration of 625.0 mg L^–1^ to each 2.0 mL of artificial wastewater. Total volumes were completed to 4 mL with ultrapure water (*V*_total_ = 4.0 mL).

### 2.7 Method Validation of the Developed Method against the UV–Vis Reference Method for Fluoride Detection

The recommended method was validated against the slightly modified UV–Vis reference method.^47^ The reference and recommended methods were compared at the desired confidence level using the *t*- and *F*-statistical tests. The preparation of solution A and solution B and the application of the proposed method are briefly described below:

Solution A: 625.0 mg L^–1^ stock solution of fluoride.
Solution B: 20.0 mL (2 × 10^–2^ M) of Fe^3+^ + 35.0 mL of 1.0% methyl salicylate + 30.0 mL of EtOH + 15.0 mL of ultrapure water.

For samples, 1.0 mL of solution B + different volumes (20.0–120.0 μL) of solution A + make-up with ultrapure water to a total volume of 2.5 mL (*V*_total_ = 2.5 mL).

For blank solution, 1.0 mL of solution B + 1.5 mL of ultrapure water (*V*_total_ = 2.5 mL).

### 2.8 Statistical Analysis

Descriptive statistical analyses were performed using Excel software (Microsoft Office 2019) for calculating the means and the standard error of the mean. Results were expressed as mean ± standard deviation (SD). Validation of the recommended method for determining the fluoride content against the UV–Vis reference method^47^ was made using the statistical tools of the same software.

## 3 Results and Discussion

### 3.1 Optimization of the Recommended Method Parameters

The figures obtained for the optimization of each parameter were formed as a result of three repetitive analyses (*N* = 3).

**Figure 1:**
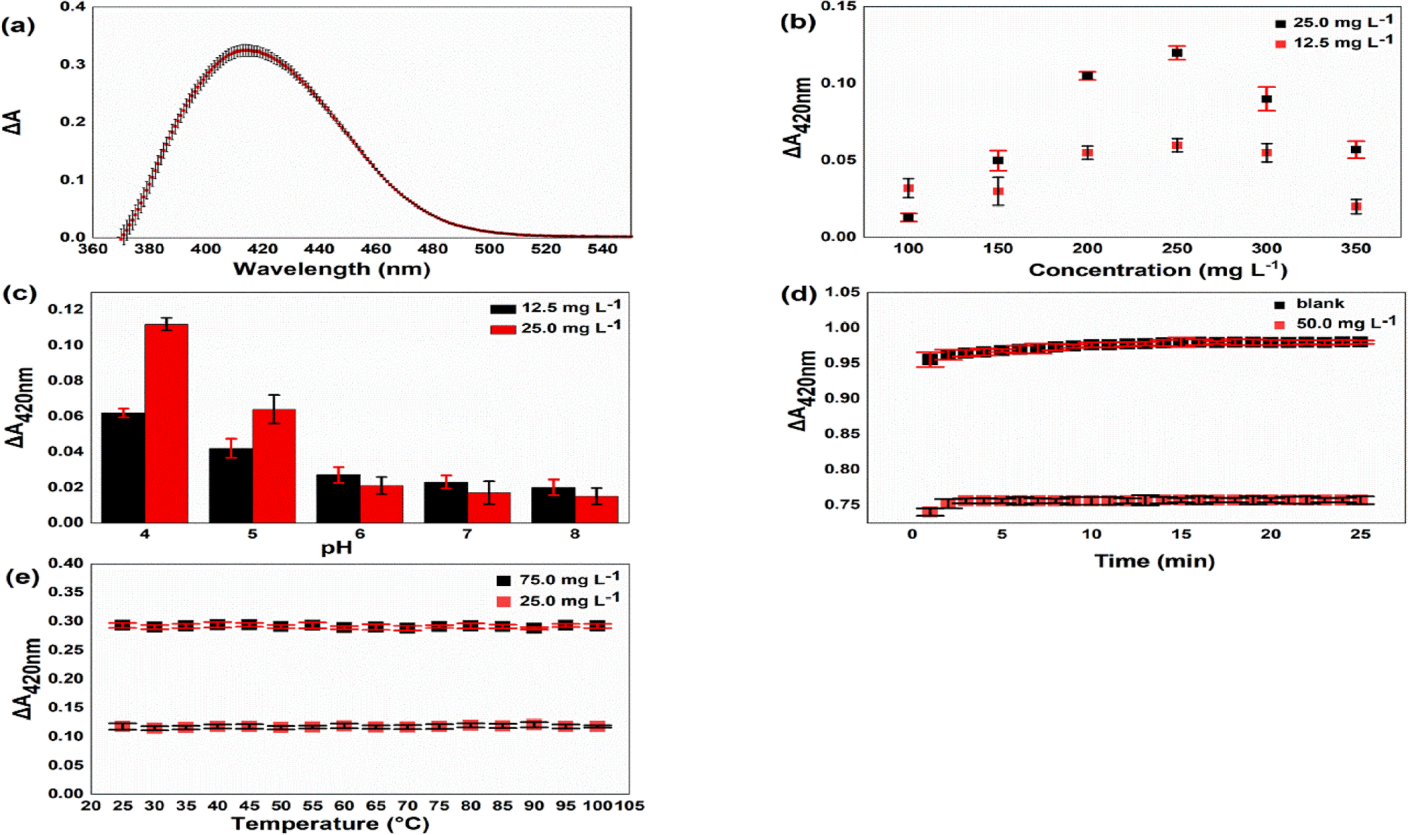
Optimization of the recommended method parameters: (a) optimal wavelength, (b) optimal Al^3+^ concentration, (c) optimal pH, (d) optimal time, and (e) optimal temperature.

To find the optimal wavelength, St. John’s wort extract (1.0 mL), pH 4.0 acetic acid-acetate buffer solution (1.0 mL, 0.1 M), 250.0 mg L^–1^ (initial conc.) Al^3+^ solution (0.5 mL), and F^–^ at 75.0 mg L^–1^ initial concentration (0.5 mL) were added to test tubes (*V*_total_ = 3.0 mL), and for the blank, 0.5 mL of ultrapure water was added instead of F^–^ solution. Then, after the blank and sample solutions were kept for 10.0 min, their absorbances were recorded against water in the wavelength range of 370–550 nm, and the wavelength at which the absorbance difference between the blank and the sample (Δ*A*) was maximum was 420 nm, as shown in Figure 1a.

To find the optimal Al^3+^ concentration, fluoride solutions at 12.5 and 25.0 mg L^–1^ initial concentrations were tested separately with Al^3+^ solutions at different concentrations. Initial concentrations of Al^3+^ ranged from 100.0 to 350.0 mg L^–1^. After adding solutions as stated below and waiting for 10.0 min, *A*_420nm_ readings were recorded against water. St. John’s wort extract (1.0 mL) + 1.0 mL of pH 4.0 acetic acid-acetate buffer solution (0.1 M) + 0.5 mL of different initial concentrations of Al^3+^ solution + 0.5 mL of F^–^ at 12.5 or 25.0 mg L^–1^ initial concentration were added to test tubes (*V*_total_ = 3.0 mL). For blank solutions, 0.5 mL of ultrapure water was added instead of F^–^ solution. The optimal initial Al^3+^ concentration was chosen as 250.0 mg L^–1^ due to the maximum Δ*A*_420nm_ readings recorded with two different concentrations of F^–^ as shown in Figure 1b.

To find the optimal pH value, studies were made in the range of pH 4.0–8.0 and also separate experiments were carried out at two different initial concentrations of fluoride as 12.5 and 25.0 mg L^–1^. After adding the solutions sequentially as stated below, they were kept for 10.0 min at RT, and the absorbance values of the blank and sample solution were read at 420 nm wavelength.

For sample solutions, add 1.0 mL of St. John’s wort extract + 1.0 mL of buffer solvents at different pH values (pH 4.0, pH 5.0, pH 6.0, pH 7.0, or pH 8.0) + 0.5 mL of 250.0 mg L^–1^ (initial conc.) Al^3+^ solution + 0.5 mL of F^–^ at initial different concentrations (12.5 or 25.0 mg L^–1^) (*V*_total_ = 3.0 mL), wait for 10.0 min at RT, and measure absorbance at λ_420 nm_ against water. For blank solution, 0.5 mL of ultrapure water was added instead of F^–^. Acetic acid–sodium acetate buffer solution (for pH 4.0 and pH 5.0), potassium hydrogen phthalate-NaOH buffer solution (for pH 6.0), ammonium acetate (for pH 7.0) buffer solution, and borax-HCl buffer solution (for pH 8.0) were used for the optimization. The absorbance differences with two different fluoride concentrations were maximum at pH 4.0, and this pH was chosen as the optimal value as shown in Figure 1c. It may be deduced that as the pH was raised above pH 4, the Al-flavonoid complex became more stable due the deprotonation of phenolic chromogen, thereby making the ligand exchange (i.e., flavonoid displacement with fluoride from the Al(III) coordination sphere) reaction more difficult for fluoride. On the other hand, as the pH was lowered below pH 4, the relative abundance of fluoride decreased because of the formation of weak acid HF and the conditional stability of the Al-flavonoid complex decreased due to the protonation of phenolic chromogen.

To select the optimal time, separate experiments were made for blank solution and 50.0 mg L^–1^ initial concentration of fluoride. For this purpose, 1.0 mL of St. John’s wort extract + 1.0 mL of pH 4.0 acetic acid-acetate buffer solution (0.1 M) + 0.5 mL of 250.0 mg L^–1^ (initial conc.) Al^3+^ solution + 0.5 mL of F^–^ at 50.0 mg L^–1^ initial concentration were added to test tubes (*V*_total_ = 3.0 mL).

For blank solution, 0.5 mL of ultrapure water was added instead of fluoride. After the solutions were added, the blank solutions and samples were incubated separately for up to 25 min at 1 min intervals, and then *A*_420 nm_ values were recorded against water. As seen in Figure 1d, the optimal time for the blank solution was 10 min, while for the samples, it was 3 min. It can be said that the interaction of Al(III) with flavonoids in the blank solution had more covalent character than the formation of the AlF_6_^3–^ complex, because the optimal times of the blank solution and samples were different. Since the absorbance readings of the blank solution and samples were recorded against water, the optimal time was chosen as 10 min for the recommended method.

To determine the optimal temperature, fluoride solutions at 25.0 and 75.0 mg L^–1^ initial concentrations were studied separately. St. John’s wort extract (1.0 mL) + 1.0 mL of pH 4.0 acetic acid-acetate buffer solution (0.1 M) + 0.5 mL of 250.0 mg L^–1^ (initial conc.) Al^3+^ solution + 0.5 mL of F^–^ at 25.0 or 75.0 mg L^–1^ initial concentration were added to test tubes (*V*_total_ = 3.0 mL). For blank solutions, 0.5 mL of ultrapure water was added instead of fluoride. The blank and sample solutions were left to stand for 10 min at different temperature intervals (25.0–100.0 °C), and then *A*_420 nm_ values were recorded against water.

Since it was observed that the recommended method was independent of temperature as seen in Figure 1e, RT was chosen as the optimal temperature for a more easily applicable method. Actually, Al(III) complexation with fluoride basically depends on electrostatic interactions (i.e., having less covalent character than that with flavonoid), and ionic complexation reactions are known to be less dependent on temperature.

### 3.2 Working Principle of the Recommended Method

**Figure 2:**
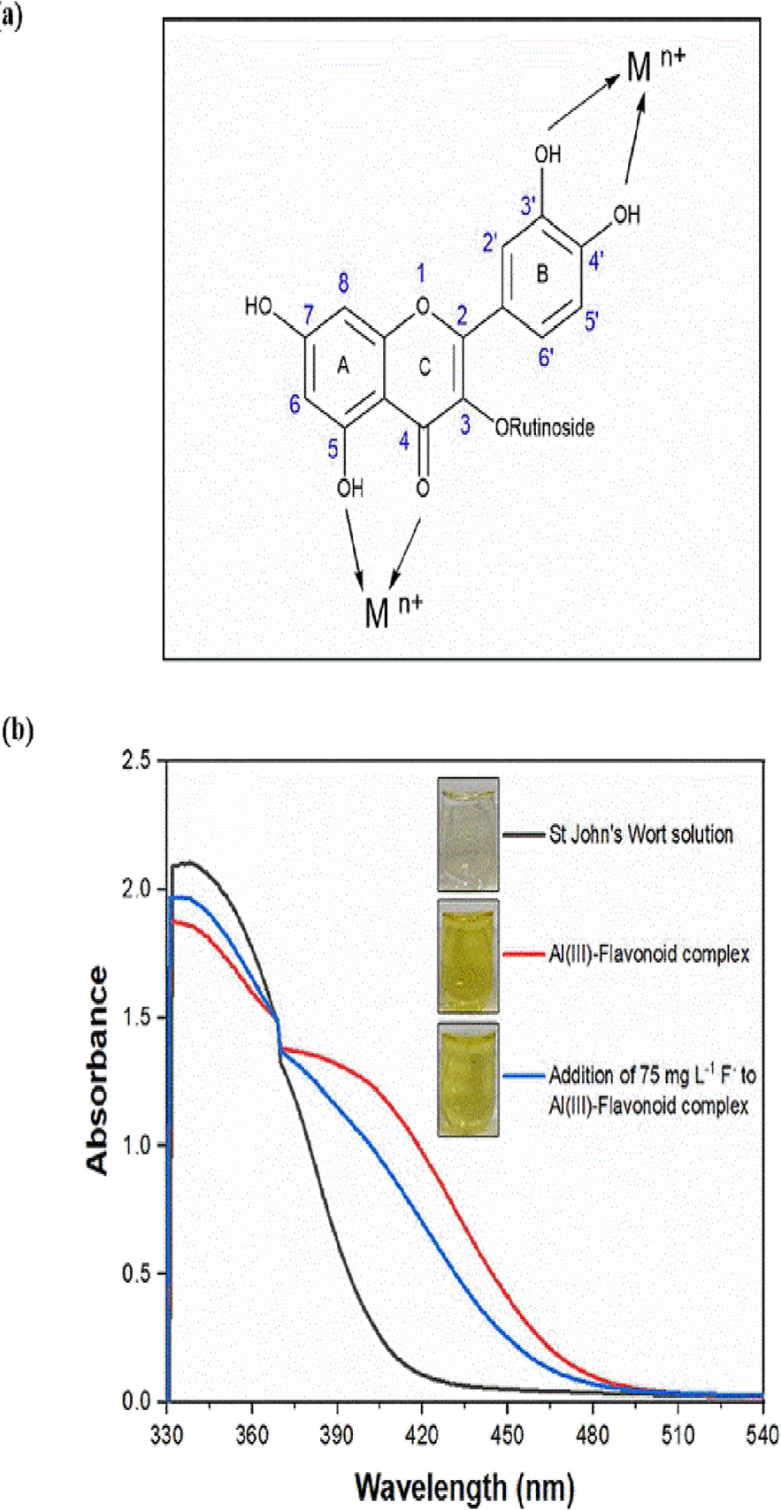
Working principle of the recommended method: (a) possible metal complexation sites of rutin; (b) representation of the visible spectrum and inset photograph of St. John’s wort extract, Al(III)-flavonoid complex, and addition of 75 mg L^–1^ (initial conc.) F^–^ to Al(III)-flavonoid complex solution.

The 3′-4′ dihydroxy group in the B ring, the 3-hydroxy or 5-hydroxy groups, and the 4-carbonyl group in the C ring are known as the three possible moieties of flavonoids that are responsible for reacting with metal ions (Figure 2a).^48^ As a result of flavonoids forming a coordination compound with metals, their absorption spectra change and a bathochromic (red) shift is observed in the UV–Vis spectrum. According to some authors, the reason for this red shift is a strong charge transfer transition from the flavonoids to the metal center, while others argue that the decrease in the HUMO-LUMO energy levels of the flavonoid molecules is greater than the charge transfer from the ligand to the center.^49^ For fluoride determination, the chromogenic agent was formed by providing a red shift in the absorption band due to the formation of the Al(III)-flavonoid complex from the St. John’s wort extract solution. Then, by adding fluoride ions to the medium, aluminum ions—capable of forming colored complexes with flavonoids—selectively form a colorless AlF_6_^3–^ complex. Therefore, a blue (hypsochromic) shift in the absorption band of flavonoids is observed (Figure 2b). Taking advantage of this phenomenon, the recommended method is based on the decolorization of the dark yellow color of the chromogenic agent in direct proportion to the amount of fluoride added to the medium. The pH of this competitive ligand exchange reaction for Al(III) was optimized by considering the relative stabilities of Al-flavonoid and Al-fluoride complexes.

### 3.3 Analytical Performance of the Recommended Method for the Determination of Fluoride

**Figure 3:**
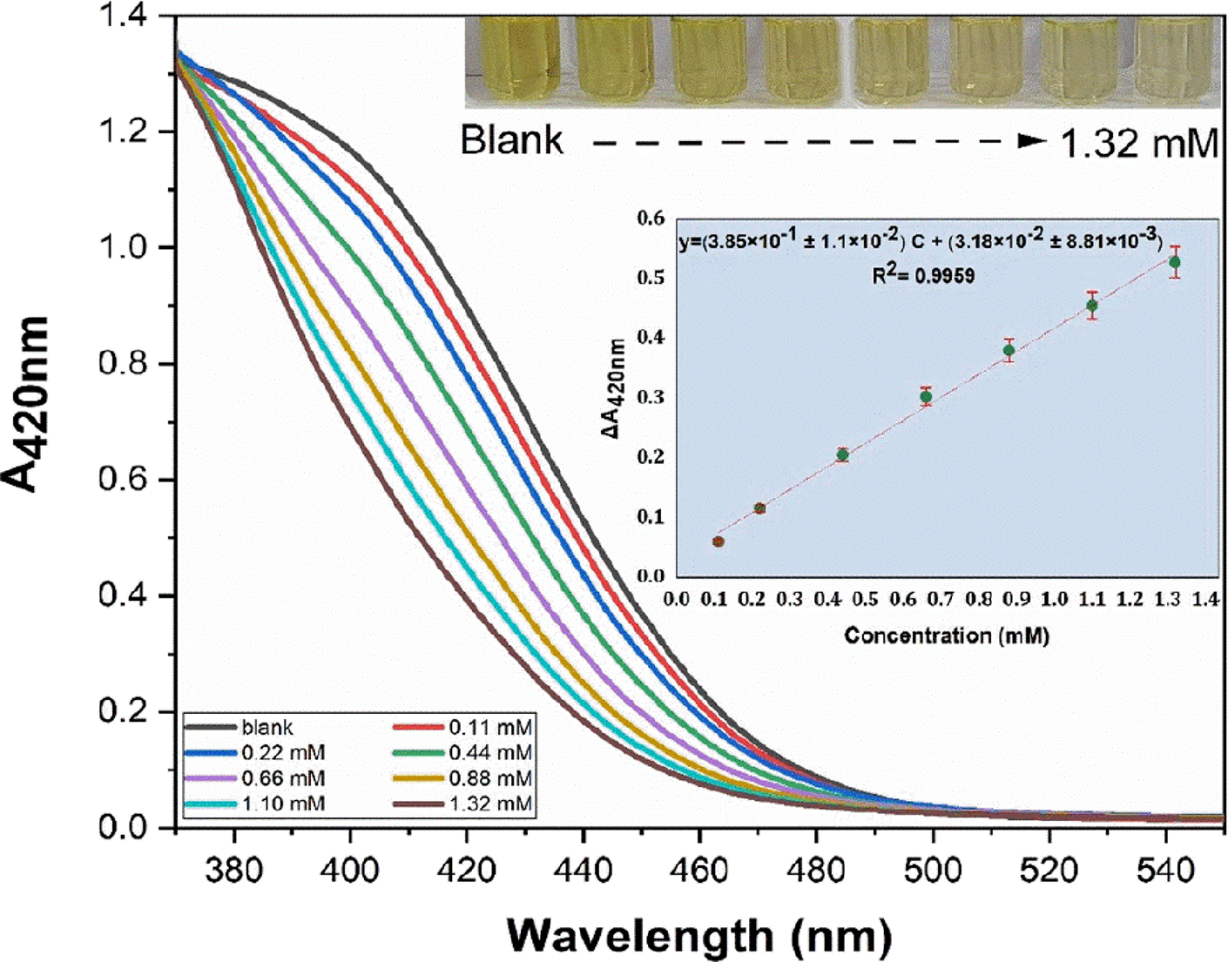
Visible spectrum of the Al(III) complex with the flavonoid (in St. John’s wort extract) containing different concentrations of fluoride in aqueous medium (inset: the calibration plot and also the photograph of the Al(III)-flavonoid complex solutions mixed with different concentrations of fluoride in ultrapure water).

It was observed that the decreasing absorbance of Δ*A*_420 nm_ ((*A*_o_ – *A*_i_)_420 nm_) was related to the increasing fluoride concentration when the proposed method was applied. In this recommended method, as the concentration of fluoride solutions increased, the decolorization (from dark yellow to light yellow) of the Al(III)-flavonoid complex was observed (Figure 3). All the absorbance measurements were recorded against water at 420 nm wavelength. The linear calibration equation was obtained with the data of Δ*A*_420 nm_ against the fluoride concentration (eq 1).

Linear calibration equation for fluoride:
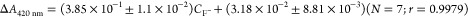
1where *C*_F^–^_ is the final concentration of fluoride (in millimoles per liter).

The linear concentration range was from 0.11 to 1.32 mM (2.0 to 25.0 mg L^–1^), covering an order of magnitude for *C*_F^–^_. In addition, the molar absorptivity (ε), limit of detection (LOD), and limit of quantification (LOQ) values were 7.67 × 10^2^ ± 2.2 × 10^1^ L mol^–1^ cm^–1^, 0.026 mM (0.5 mg L^–1^), and 0.079 mM (1.5 mg L^–1^), respectively. The limit of detection was found in millimole per liter units (LOD = 3σ_bl_/*m*, with σ_bl_ denoting the standard deviation of a blank and *m* showing the slope of the calibration line). The coefficients of variation (CVs) of intra- and inter-assay for fluoride were 2.17 and 2.59%, respectively (*N* = 5), showing that the recommended method has good precision. The LOD value is below the permissible limit of fluoride in water (0.079 mM, 1.5 mg L^–1^) by WHO^50^ and also enforceable (0.22 mM, 4.0 mg L^–1^) and non-enforceable (secondary, 0.11 mM (2.0 mg L^–1^)) limits by EPA.^51,52^

In addition, the recommended method and previously published fluoride determination methods were compared with respect to certain parameters such as solution medium, linear concentration range, LOD values, and real sample application in Table 1.

**Table 1:**
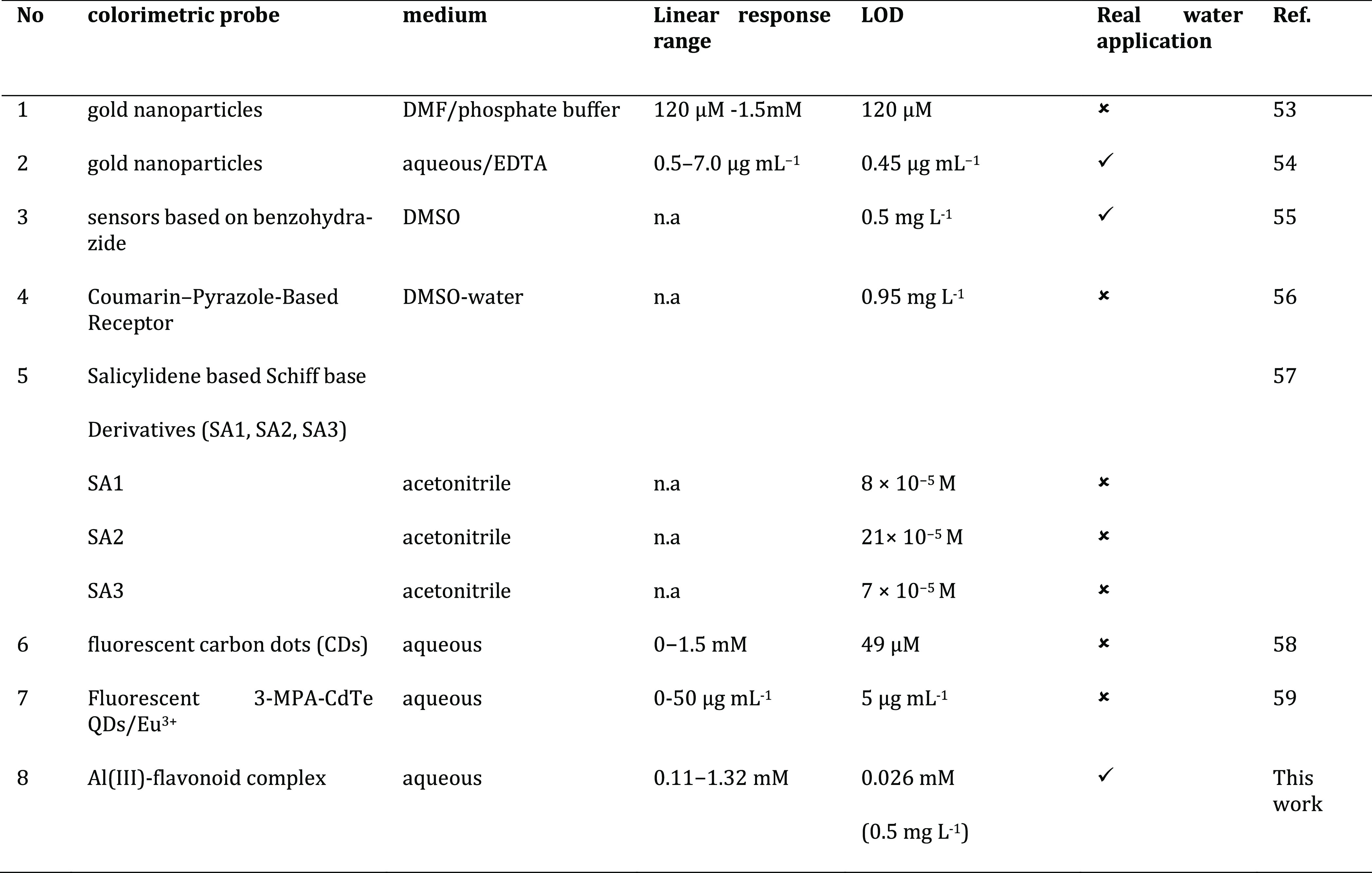
Comparison of Analytical Performances of the Optical Detection Methods for F^–^a^53−59^

a n.a: not available; SA1: salicylaldehyde-*o*-aminophenol; SA2: 3,5-dimethyl-salicylaldehyde-*o*-aminophenol; SA3: 3,5-dichloro-salicylaldehyde-*o*-aminophenol; MPA: 3-mercaptopropanoic acid; QDs: quantum dots.

### 3.4 Investigation of Interference Effect of Common Ions

**Figure 4:**
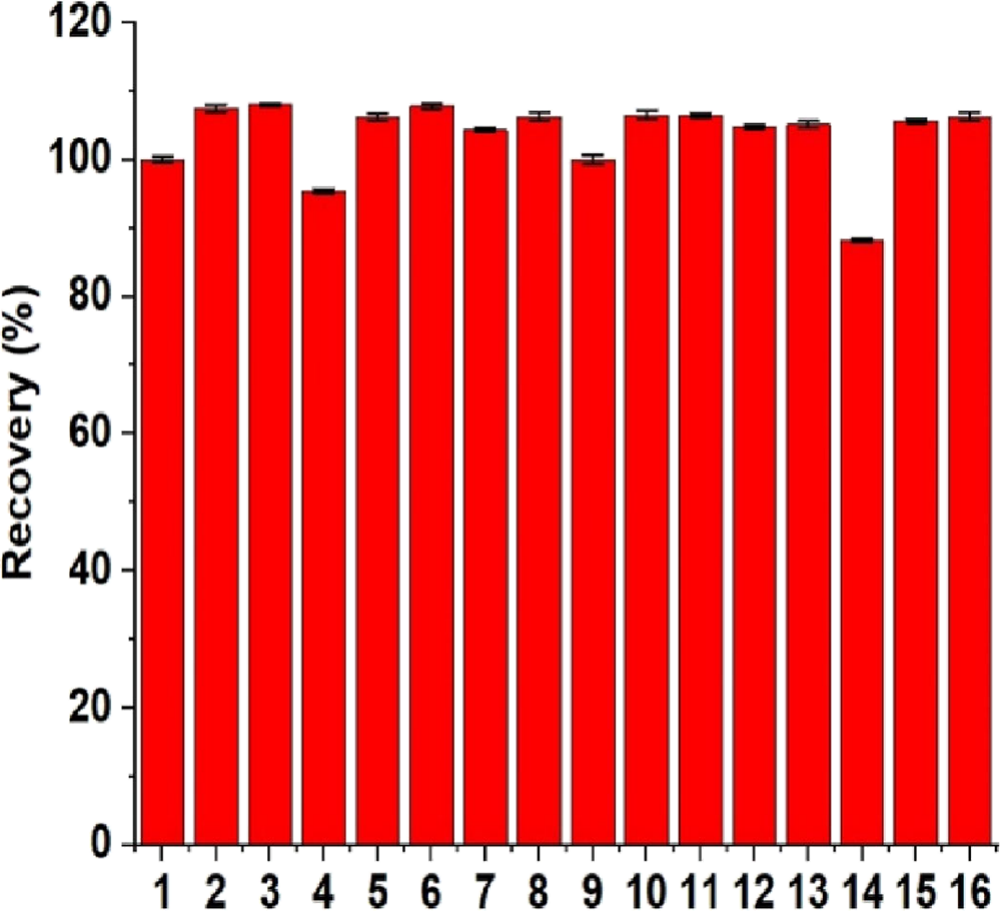
Response of fluoride (1) and possible interferent species {K^+^ (2), NH_4_^+^ (3), Ag^+^ (4), Ca^2+^ (5), Mg^2+^ (6), Mn^2+^ (7), [(Fe^2+^ + EDTA] (8), [(Fe^3+^ + EDTA] (9), Cl^−^ (10), Br^−^ (11), I^−^ (12), NO_3_^–^ (13), HCO_3_^–^ (14), SO_4_^2–^ (15) and [PO_4_^3–^ + 3Ag^+^ → Ag_3_(PO_4_)↓] (16)} in the presence of fluoride at initial concentration of 50 mg L^–1^.

The possible interference effects of common anions (Cl^–^, Br^–^, I^–^, NO_3_^–^, HCO_3_^–^ SO_4_^2–^, and PO_4_^3–^) and cations (K^+^, NH_4_^+^, Ag^+^, Ca^2+^, Mg^2+^, Mn^2+^, Fe^2+^, and Fe^3+^) present in water on the proposed method were investigated. The interferences of Fe^2+^ and Fe^3+^ on the method were eliminated using Na_2_EDTA as a specific masking agent without affecting aluminum ions that complexed with the flavonoid. PO_4_^3–^ interference was removed by the precipitation method with AgNO_3_. The interference analysis was performed using solutions of fluoride at an initial concentration of 50.0 mg L^–1^ with different mass ratios of common water ions such as Cl^–^, Br^–^, I^–^, NO_3_^–^, HCO_3_^–^, SO_4_^2–^, PO_4_^3–^, K^+^, NH_4_^+^, Ag^+^, Ca^2+^, Mg^2+^, Mn^2+^, Fe^2+^, and Fe^3+^ (i.e., 1-, 30-, 40-, 100-, 250-, and 500-fold of fluoride). The interference effects of anions and cations are represented in Table 2, and the fluoride recovery (%) values were found between 88.2 and 108.0% (Figure 4).

**Table 2:** F^–^ Recoveries (%) for Application of the Proposed Method to Ionic Species Commonly Found in Water at Different Mass Ratios to F^–^

interferent	error (%)	mass ratio (F^–^:ions (w/w))
Cl^–^	+6.50	250
Br^–^	+6.50	500
I^–^	+4.70	500
NO_3_^–^	+5.10	500
HCO_3_^–^	–11.8	9
SO_4_^2–^	+5.60	40
PO_4_^3–^	before precipitation with Ag^+^: **+20.9**	1
	after precipitation with Ag^+^: +6.30	
K^+^	+7.52	250
NH_4_^+^	+8.00	100
Ag+	–4.56	100
Ca^2+^	+6.23	100
Mg^2+^	+7.80	30
Mn^2+^	+4.30	100
Fe^2+^	before EDTA masking: −99.99	1
	after EDTA masking: +6.29	
Fe^3+^	before EDTA masking: −99.98	1
	after EDTA masking: 0.00	

The initial concentration of Na_2_EDTA, forming a complex with the interfering iron ions (Fe^2+^ and Fe^3+^) but not interacting with aluminum in the Al(III)-flavonoid complex, was investigated. For this purpose, a maximal initial concentration of 300.0 mg L^–1^ Na_2_EDTA was added instead of fluoride, and no change was observed in the absorbance of the blank solution. The interference effect of Fe^2+^ and Fe^3+^ was easily eliminated by using Na_2_EDTA as a masking agent, which was added at different mass ratios for each valency of iron [metal–EDTA ratio: 1:3 (w/w) for Fe^2+^ and 1:6 (w/w) for Fe^3+^] to 1:1 iron:fluoride solutions before applying the recommended method. Since ferric ions had a higher affinity for fluoride than ferrous ions in accordance with crystal field theory, they required a higher concentration of the chelating agent (EDTA) for sufficient masking. During this procedure, the Al(III)-flavonoid complex was not affected because Al(III) was already bound in a stable complex, and Al(III) chelation with EDTA needs temperature and time (i.e., Al(III) has slow kinetics due to its noble gas electronic configuration).

Elimination of the interference effect of phosphate is based on the principle of removing it from solution by precipitation with silver ions in acidic medium (details are given in Section 2.5). The proposed method was applied to solutions having initial concentrations of 50.0 mg L^–1^ F^–^, 50.0 mg L^–1^ PO_4_^3–^, and 50.0 mg L^–1^ Ag^+^ mixture obtained in the last step.

### 3.5 Application of the Recommended Method to Real Water Samples

The concentrations in mg L^–1^ of the ions specified in the purchased mineral water were as follows: F^–^: 2.11; HCO_3_^–^: 1062; SO_4_^2–^: 24.77; Cl^–^: 59.12; Ca^2+^: 154; K^+^: 35; Mg^2+^: 57; Fe^2+^: <0.02; Na^+^: 164. The final concentration of fluoride in the mineral water after the necessary pH adjustment and dilution steps (i.e., during the application of the recommended method) was 0.281 mg L^–1^.

The proposed method was applied to mineral water with standard additions, with a final concentration range of 4.447–16.947 mg L^–1^ (details are given in Section 2.6). Fluoride recoveries (%) and RSD % were found to be between 99.8 and 102.9% and between 0.60 and 4.44%, respectively, as shown in Table 3.

**Table 3:** Application of the Recommended Method to Real Water Samples for the Determination of F^–^

sample	F^–^ found (mg L^–1^)	F^–^ added (mg L^–1^)	F^–^ (spiked + sample water) expected (mg L^–1^)	F^–^ (spiked + sample water) found (mg L^–1^)	recovery (%)	RSD (%)
mineral water	0.281	4.166	4.447	4.452	100.1	4.44
		8.333	8.614	8.863	102.9	1.28
		12.500	12.781	12.750	99.8	0.77
		16.666	16.947	16.949	100.0	0.60
						
artificial wastewater	4.166	4.166	8.332	8.467	101.6	1.39
		8.333	12.499	12.559	100.5	0.21
		12.500	16.666	16.899	101.4	0.92
		16.666	20.832	19.506	93.6	1.15

According to the literature,^60^ artificial wastewater was prepared with a mixture of initial concentrations of 100.0 mg L^–1^ B (H_3_BO_3_ was used as a boron source) and 300.0 mg L^–1^ F^–^. Concrete sludge is an industrial waste slurry containing hydrated cement, aggregates, and water, and its wastewater may contain both boron and fluoride.^60^ The proposed method was applied to artificial wastewater with standard additions at a final concentration range of 4.166–16.666 mg L^–1^ (details are given in Section 2.6). Fluoride recoveries (%) and RSD % were found to be between 93.6 and 101.6% and between 0.21 and 1.39%, respectively, as shown in Table 3.

### 3.6 Validation of the Recommended Fluoride Determination against the UV–Vis Reference Method

The recommended method was validated against the UV–Vis reference method^47^ after slight modifications (details are given in Section 2.7). The final fluoride concentration range of 5–30 mg L^–1^ was determined with the UV–Vis reference method to form the calibration equation, which was:
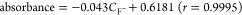
for fluoride, where *C*_F^–^_ is the final concentration of fluoride in mg L^–1^.

After preparing fluoride solutions to have an initial concentration of 75 mg L^–1^ in the artificial wastewater from five different mixtures with initial concentrations of 300 mg L^–1^ F^–^ and 100 mg L^–1^ B solutions (H_3_BO_3_ as the boron source), absorbance readings were recorded for the recommended method and the reference method (final conc. of F^–^ was 12.5 mg L^–1^ when both methods were applied). The mean of the absorbance readings of three consecutive samples was used for each calculation (*N* = 3). The results showed no significant difference in precision and accuracy between the two methods. The recommended method was validated against the UV–Vis reference method at the 95% confidence level using Student’s *t*- and *F*-tests. Statistical parameters of the recommended method and the UV–Vis reference method are depicted in Table 4.

**Table 4:** Statistical Comparison of the Recommended Method with UV–Vis Reference Method for Fluoride Determination

method	mean conc. (mg L^–1^)	SD (σ)	*S*^a^^,^^b^	*t*^a^^,^^b^	*t*_tablo_^b^	*F*^b^	*F*_tablo_^b^
recommended method	12.621	0.134					
UV–Vis reference method	12.551	0.151	0.169	0.291	2.306	1.591	6.39

a *S*^2^ = ((*n*_1_ – 1)*s*_1_^2^ + (*n*_2_ – 1)*s*_2_^2^)/(*n*_1_ + *n*_2_ – 2) and *t* = (*a̅*_1_ – *a̅*_2_)/(*S*(1/*n*_1_ + 1/*n*_2_)^1/2^), where *S* is the pooled standard deviation, *s*_1_ and *s*_2_ are the standard deviations of the two populations with sample sizes of *n*_1_ and *n*_2_, and *a̅*_1_ and *a̅*_2_ are sample means (*t* has (*n*_1_ + *n*_2_ – 2) degrees of freedom); here, *n*_1_ = *n*_2_ = 5.

b Statistical comparison made on paired data produced with the proposed and reference methods; results given only on the row of the reference method.

## 4 Conclusions

In this study, our main aim was to develop a simple and inexpensive fluoride determination method based on the principles of green analytical chemistry, free from toxic solvents. This method was eco-friendly without complicated steps and utilized a natural extract in a green chromogenic method that meets today’s requirements. For this purpose, St. John’s wort was chosen as a natural sensing reagent for fluoride determination by utilizing the Al(III)-flavonoid metal complex formation due to the strong charge transfer interaction of flavonoids with metal ions and/or the decrease in HUMO-LUMO energy levels of flavonoids, which is more effective than ligand-to-metal charge transfer. It is clear that the LOD value (0.5 mg L^–1^) of the recommended method meets the required critical values of WHO (1.5 mg L^–1^) and EPA (4 mg L^–1^ for the non-enforceable standard and 2 mg L^–1^ for the secondary standard) for the fluoride content of water samples. The possible interferences of ionic species commonly found in water were investigated for establishing the selectivity of the recommended method for the analyte. While Na_2_EDTA was used to selectively remove the interference effects of Fe^2+^ and Fe^3+^, the precipitation method was applied in acidic medium to eliminate the interference of PO_4_^3–^ using Ag^+^ ions as a precipitation agent. The recommended method was applied to water samples (mineral water and artificial wastewater from concrete sludge) and also validated against a reference UV–Vis reference method at the 95% confidence level.
